# Acute Sleep Loss Increases Circulating Morning Levels of Two MicroRNAs Implicated in Neurodegenerative Disease in Healthy Young Men

**DOI:** 10.1111/jcmm.70523

**Published:** 2025-04-07

**Authors:** Lei Zhang, Anastasia Grip, Daisy Hjelmqvist, Christian Benedict, Luiz Eduardo Mateus Brandão, Jonathan Cedernaes

**Affiliations:** ^1^ Department of Medical Sciences Uppsala University Uppsala Sweden; ^2^ Department of Medical Cell Biology Uppsala University Uppsala Sweden; ^3^ Department of Pharmaceutical Biosciences Uppsala University Uppsala Sweden

**Keywords:** Alzheimers disease, microRNA, neuronal inflammation, simulated shift work, sleep, total sleep deprivation

## Abstract

Chronic sleep disruption and shift work elevate the risk of neurodegeneration and Alzheimer's disease (AD). While disrupted sleep affects canonical AD biomarkers, its impact on other mechanisms, such as circulating microRNAs (miRNAs), remains less understood. Therefore, we here examined the effects of overnight wakefulness on plasma levels of several miRNAs implicated in neurodegeneration and AD, as well as in sleep and circadian regulation—namely miR‐127‐3p, miR‐132‐3p, and miR‐142‐3p. Following a baseline period in each highly controlled in‐lab session, in total 15 healthy normal‐weight young men underwent two conditions on separate occasions, in randomised order: a night of normal sleep, and a night of sustained wakefulness. After overnight wakefulness, morning plasma levels of miR‐127‐3p and miR‐142‐3p were significantly elevated compared with post‐sleep levels. These changes were not associated with the significant increase in self‐reported morning stress levels observed after wakefulness compared with sleep. This study is the first to demonstrate that a single night of wakefulness—mimicking overnight shift work—significantly elevates circulating levels of miR‐127‐3p and miR‐142‐3p in humans. These findings, though based on a limited sample size, suggest a potential molecular link between sleep loss and neurodegeneration, warranting further investigation.

**Trial Registration:** Clinical Trial number: NCT01800253; www.clinicaltrials.gov

Recurrent and chronic sleep disruption (SD)—including shift work and the sleep disorder insomnia—are highly prevalent today and are associated with an increased risk of Alzheimer's disease (AD). Indeed, a meta‐analysis found that subjective sleep issues confer a ~ 55% higher risk of AD [1]. Given that AD takes decades to develop, it is imperative to uncover the mechanisms by which disrupted and misaligned sleep (as in shift work) can promote the pathogenesis of AD. Whereas prior studies have focused on the impact of SD on canonical AD biomarkers—such as Tau—other putative biomarkers, such as microRNAs (miRNAs), may also impact neurodegenerative processes in humans.

At the mechanistic level, sleep has been found to increase CSF flow in humans and mice to promote the glymphatic clearance of neurotoxins associated with AD pathogenesis, such as amyloid β. Extended wakefulness in sleep loss sustains higher neuronal activity, which contributes to increased central nervous system (CNS) production of such AD biomarkers. However, far less is known about how cellular pathways that impact neuronal health—and which may be regulated, for example, by specific miRNAs—are impacted by SD in humans.

MiRNAs are short (18–25 nucleotides) non‐coding RNAs that regulate gene expression at the post‐transcriptional level. miRNAs can exert their role in a paracrine fashion in remote tissues, such as the CNS. Thanks to their stability and accessibility in biofluids such as blood, miRNAs have garnered interest as potential biomarkers for neurodegenerative diseases such as AD [2]. Gaining further insight into how blood‐borne miRNAs may be involved in AD pathogenesis is therefore of importance.

Although the regulatory mechanisms of miRNAs can differ across studies and sample types [2], miR‐127‐3p, miR‐132‐3p, and miR‐142‐3p have been consistently implicated in AD across independent studies, demonstrating higher specificity compared with other miRNAs (see Table S1). In a meta‐analysis, upregulation of miR‐127‐3p in serum has been associated with inflammation and apoptosis, and this miRNA is significantly differentially expressed in patients with AD [3]. miR‐132 is involved in neurodegeneration and AD, by for example, regulating neurogenesis and tau phosphorylation. Rescuing miR‐132 levels mitigated adult hippocampal neurogenesis and memory deficits in a mouse AD model [4]. miR‐142‐3p regulates neuroinflammation and metabolic pathways, and may serve as a potential biomarker of neurodegeneration [5]. Furthermore, a genetic variant that reduces the expression of miR‐142 is associated with lower risk of AD [6]. Compared with baseline values, extended wakefulness for 40 h was found to upregulate afternoon serum levels of miR‐127—with normalisation after subsequent recovery sleep [7]. Currently, little is known about how more common types of SD, that is, wakefulness restricted to one overnight period (common in, for example, shift workers), impact circulating levels of miRNAs associated with neurodegeneration and AD.

We therefore assessed morning levels of miR‐127‐3p, miR‐132‐3p, and miR‐142‐3p in blood from a randomised crossover study comparing overnight wakefulness with a full night of sleep. Given that sleep disruption may promote pathways involved in early AD pathogenesis, we hypothesised that overnight wakefulness would increase circulating plasma levels of these miRNAs in healthy individuals. As miRNAs have been implicated in the response to acute psychological stress in humans, we also explored whether changes in circulating miRNA levels correlated with increased subjective stress in response to overnight wakefulness (see Supplement for more details).

In the 15 normal‐weight young men who completed both sessions successfully, we found that plasma levels of miR‐127‐3p and miR‐142‐3p were significantly higher after overnight wakefulness compared with after a night of sleep (Wilcoxon signed rank test: *p* = 0.0245 and *p* = 0.0256, respectively, Figure 1). These results largely held up in paired 10,000‐iteration permutation tests, although miR‐127‐3p exhibited a weakened association (miR‐127‐3p: *p* = 0.095; miR‐142‐3p: *p* = 0.020). Higher values after wakefulness versus sleep were observed in ~71% and ~67% of participants, for miR‐127‐3p and miR‐142‐3p, respectively. Levels of miR‐132‐3p were, however, not significantly impacted by overnight wakefulness (*p* = 0.46 by Wilcoxon; *p* = 0.385 by permutation).

**FIGURE 1:**
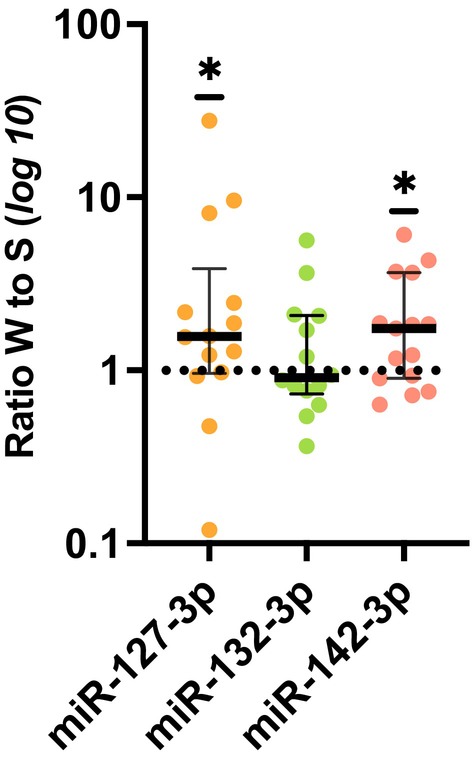
Changes in relative morning plasma levels of miR‐127‐3p, miR‐132‐3p, and miR‐142‐3p, in response to overnight wakefulness compared with normal sleep. Shows subject‐specific changes, calculated as the fold change in relative morning miRNA values after overnight wakefulness (W), compared with the morning after a full night of sleep (S). *Signifies *p* < 0.05, based on a Wilcoxon signed‐rank test results. For visualisation purposes, log_10_‐transformed values are shown; the figure shows the median as a thick solid line for each miRNA, and the 25% and 75% percentile are shown as the lower and upper thinner solid lines, respectively. *N* = 14 within‐subject pairs for miR‐127‐3p and miR‐132‐3p (undetectable qPCR values for one subject for each of these miRNAs); *n* = 15 within‐subject pairs for miR‐142‐3p.

While subjective ratings of stress at 0800 h were elevated about twofold after overnight wakefulness compared with after sleep (mean ± S.E.M. change: +108 ± 49%, *p* = 0.0467), this induction was not correlated with the wake‐to‐sleep change in levels of miR‐127‐3p (*r* = 0.21, *p* = 0.49) or miR‐142‐3p (*r* = 0.42, *p* = 0.16).

Dynamics in miRNA expression during the progression of neurodegenerative conditions such as AD may be key for understanding variability across previously described different sample types. Our finding of upregulated miR‐142‐3p in plasma following acute sleep deprivation may be relevant given the upregulation of this miRNA in the plasma of cognitively normal but amyloid‐positive (CN‐Aβ+) individuals. Plasma levels of miR‐142‐3p are progressively reduced from mild cognitive impairment (MCI) to overt AD, suggesting a potential involvement in early AD neurodegenerative processes [5]. Notably, miR‐142‐3p expression is one of the miRNAs that is significantly increased by chronological age in peripheral blood [8] and an *MIR142*‐proximal genetic polymorphism (rs2632516) was borderline significant for late‐onset Alzheimer's disease in a genome‐wide association meta‐analysis (*p* = 5.3 × 10^−8^; genome‐wide significance at 5 × 10^−8^) [9]. As sleep typically deteriorates with age, and as sleep disruption is common in AD, the potential sleep‐centred role of miR‐142 in AD pathogenesis may warrant further investigation.

A prior study found that several miRNAs can be induced by psychological stress in humans [10]. In our study, it is possible that physiological rather than psychological stress contributed to the extended wakefulness‐induced increase in miRNA levels. A rodent model of hypoxic injury resulted in autophagic apoptosis and neurological impairments that were partly alleviated by ablating miR‐127‐3p expression [11]. Given that overnight wakefulness increases circulating levels of biomarkers that indicate neuronal injury in young healthy individuals [12], it can be speculated whether suppression of miR‐127‐3p could possibly be helpful in alleviating such neuronal stress in humans.

Despite stemming from a highly controlled randomised trial, the present study has several limitations. Blood levels may not reflect miRNA levels or activity within the CNS, and none of the investigated miRNAs are brain‐enriched. Therefore, studies are needed to establish whether sleep loss‐induced changes primarily reflect peripheral tissue responses. Such responses may nonetheless impact brain health. For example, miR‐142‐3p can modulate the molecular clock; future studies should investigate how interventions that induce circadian misalignment may modulate miRNA levels and downstream actions. It remains to be established whether our results would differ in females, older individuals, other ethnicities, or in individuals at greater risk of brain pathology (such as shift workers). Unlike a prior 40‐h wakefulness study, recovery sleep was not included. While overnight wakefulness reflects typical shift worker conditions, the time needed for miRNA levels and downstream effects to normalise after recovery sleep remains unclear. As such, any potential long‐term impact of chronically disrupted sleep–wake patterns—which may also impact levels of miR‐132‐3p—remains to be established.

This study is the first to reveal that overnight wakefulness significantly impacts circulating levels of miR‐127‐3p and miR‐142‐3p in humans. These findings—which, due to their sample size, should be seen as hypothesis‐generating—may guide further investigations into the molecular mechanisms through which chronic sleep disruption promotes neurodegeneration and AD. Of note, we herein observed high variability in the wakefulness‐mediated induction of the studied miRNAs. If individuals with larger responses would show a steeper cognitive decline from chronic sleep disruption, these miRNAs may possibly be useful as risk biomarkers for MCI and/or AD.

## Author Contributions

**Lei Zhang:** data curation (equal), formal analysis (supporting), methodology (supporting), software (equal), visualization (equal), writing – original draft (supporting), writing – review and editing (equal). **Anastasia Grip:** data curation (supporting), formal analysis (supporting), writing – review and editing (supporting). **Daisy Hjelmqvist:** formal analysis (supporting), investigation (equal), methodology (supporting), software (supporting), validation (supporting), writing – review and editing (supporting). **Christian Benedict:** conceptualization (supporting), funding acquisition (equal), project administration (supporting), resources (supporting), writing – review and editing (supporting). **Luiz Eduardo Mateus Brandão:** formal analysis (supporting), investigation (supporting), methodology (supporting), supervision (supporting), writing – review and editing (supporting). **Jonathan Cedernaes:** conceptualization (lead), data curation (equal), formal analysis (lead), funding acquisition (equal), investigation (equal), methodology (lead), project administration (lead), resources (lead), software (supporting), supervision (lead), validation (supporting), visualization (equal), writing – original draft (lead), writing – review and editing (equal).

## Disclosure

The authors have nothing to report.

## Conflicts of Interest

The authors declare no conflicts of interest.

## Supporting information


Data S1.

## Data Availability

Anonymised data underlying this article will be shared on reasonable request to qualified researchers from accredited academic institutions.

## References

[1] O. M. Bubu , M. Brannick , J. Mortimer , et al., “Sleep, Cognitive Impairment, and Alzheimer's Disease: A Systematic Review and Meta‐Analysis,” Sleep 40, no. 1 (2017): zsw032. 10.1093/sleep/zsw032 28364458

[2] S. Herrera‐Espejo , B. Santos‐Zorrozua , P. Álvarez‐González , E. Lopez‐Lopez , and Á. Garcia‐Orad , “A Systematic Review of MicroRNA Expression as Biomarker of Late‐Onset Alzheimer's Disease,” Molecular Neurobiology 56, no. 12 (2019): 8376–8391. 31240600 10.1007/s12035-019-01676-9

[3] P. Takousis , A. Sadlon , J. Schulz , et al., “Differential Expression of microRNAs in Alzheimer's Disease Brain, Blood, and Cerebrospinal Fluid,” Alzheimer's & Dementia 15, no. 11 (2019): 1468–1477. 10.1016/j.jalz.2019.06.4952 31495604

[4] H. Walgrave , S. Balusu , S. Snoeck , et al., “Restoring miR‐132 Expression Rescues Adult Hippocampal Neurogenesis and Memory Deficits in Alzheimer's Disease,” Cell Stem Cell 28, no. 10 (2021): 1805–1821. 34033742 10.1016/j.stem.2021.05.001

[5] D. Guévremont , H. Tsui , R. Knight , et al., “Plasma microRNA Vary in Association With the Progression of Alzheimer's Disease,” Alzheimer's & Dementia: Diagnosis, Assessment & Disease Monitoring 14, no. 1 (2022): e12251. 10.1002/dad2.12251 PMC8817674 35141392

[6] M. Ghanbari , S. T. Munshi , B. Ma , et al., “A Functional Variant in the miR‐142 Promoter Modulating Its Expression and Conferring Risk of Alzheimer Disease,” Human Mutation 40, no. 11 (2019): 2131–2145. 31322790 10.1002/humu.23872

[7] S. Weigend , S. C. Holst , J. Meier , M. Brock , M. Kohler , and H.‐P. Landolt , “Prolonged Waking and Recovery Sleep Affect the Serum MicroRNA Expression Profile in Humans,” Clocks & Sleep 1, no. 1 (2018): 75–86, 10.3390/clockssleep1010008. 33089155 PMC7509676

[8] T. Huan , G. Chen , C. Liu , et al., “Age‐Associated Micro RNA Expression in Human Peripheral Blood Is Associated With All‐Cause Mortality and Age‐Related Traits,” Aging Cell 17, no. 1 (2018): e12687, 10.1111/acel.12687. 29044988 PMC5770777

[9] B. W. Kunkle , B. Grenier‐Boley , R. Sims , et al., “Genetic Meta‐Analysis of Diagnosed Alzheimer's Disease Identifies New Risk Loci and Implicates Aβ, Tau, Immunity and Lipid Processing,” Nature Genetics 51, no. 3 (2019): 414–430, 10.1038/s41588-019-0358-2. 30820047 PMC6463297

[10] C. Wiegand , P. Heusser , C. Klinger , et al., “Stress‐Associated Changes in Salivary microRNAs Can Be Detected in Response to the Trier Social Stress Test: An Exploratory Study,” Scientific Reports 8, no. 1 (2018): 7112. 29740073 10.1038/s41598-018-25554-x PMC5940676

[11] Z. B. Zhang , L. L. Xiong , L. L. Xue , et al., “MiR‐127‐3p Targeting CISD1 Regulates Autophagy in Hypoxic‐Ischemic Cortex,” Cell Death & Disease 12, no. 3 (2021): 279. 33723216 10.1038/s41419-021-03541-x PMC7961148

[12] C. Benedict , J. Cedernaes , V. Giedraitis , et al., “Acute Sleep Deprivation Increases Serum Levels of Neuron‐Specific Enolase (NSE) and S100 Calcium Binding Protein B (S‐100B) in Healthy Young Men,” Sleep 37, no. 1 (2014): 195–198. 24470708 10.5665/sleep.3336 PMC3902870

