# Supplementary material for: Acute Sleep Loss Increases Circulating Morning Levels of Two MicroRNAs Implicated in Neurodegenerative Disease in Healthy Young Men
Source: J Cell Mol Med. 2025 Apr 7;29(7):e70523. doi: 10.1111/jcmm.70523 (PMC11975503; doi:10.1111/jcmm.70523)
Supplement: Supplementary file 1 — Data S1. [file JCMM-29-e70523-s001.docx]

## Supplemental Methods.

## Experimental design

This study was a randomized crossover within-subject design conducted at the Uppsala Biomedical Centre, Sweden. The study was approved by the Regional Ethical Review Board in Uppsala (EPN 2012/477/1). All participants provided written and oral informed consent before participating in the study.

15 participants completed both study intervention conditions, i.e., overnight wakefulness and a normal night of sleep, with study conditions carried out in a randomized crossover design, with a washout period of at least 4 weeks between the two sessions. For each experimental session, participants arrived in the late afternoon (day 0) and remained under constant supervision until the session concluded (approximately 42 hours in total). Throughout their stay, participants were confined to their rooms to avoid physical exertion.

During the baseline night (22:30 to 07:00 on Day 0), all participants slept in a standardized environment in complete darkness. On the following day, participants consumed three standardized isocaloric meals (identical meals and matched timing across the two experimental conditions; caloric requirements were calculated using the Harris-Benedict equation). Meal consumption was strictly monitored by the research staff to ensure that the participants consumed each meal in its entirety. Two standardized 15-minute walks under direct experimental supervision were provided. During the remaining in-lab time, participants could engage in sedentary activities in their designated rooms.

The experimental intervention night occurred from 22:30 to 07:00 on the second day, i.e., following the baseline period (one day and one night of sleep prior to each of the two conditions). For this second night in the normal sleep condition, participants slept undisturbed in complete darkness. For this second night in the overnight wakefulness condition, participants remained awake throughout the night under continuous monitoring, and the room light intensity was maintained at about 300 lux. Participants were required to remain in a semi-recumbent position to minimize physical activity and were provided 1.5 dl of water every two hours, with additional water available upon request. No food intake was allowed during this period.

At approximately 07:30 following the experimental night, blood samples were collected from participants and then centrifuged at 3500 rpm for 15 minutes at 4°C to isolate plasma, which was stored at −80°C until sample processing.

At 08:00, participants completed a subjective stress assessment using a Visual Analogue Scale (VAS). Stress levels were rated on a scale from 0 to 100 mm, where 0 indicated “not stressed at all” and 100 indicated “very stressed.”

## miRNA isolation and qPCR

Circulating miRNAs were isolated from ~150 µL plasma from each sample, using the Qiagen miRNeasy Serum/Plasma Advanced Kit (#217204). miRNAs were quantified following initial cDNA synthesis (TaqMan™ Advanced miRNA cDNA Synthesis Kit, #A28007, ThermoFisher), using the TaqMan™ Fast Advanced Master Mix for qPCR (#4444963, ThermoFisher).

Taqman probes specific to miR-127-3p, miR-132-3p and miR-142-3p were used according to the manufacturer’s instructions (#A25576, ThermoFisher). For sample normalization against a housekeeping miRNA, relative expresssion levels of miR-23a were used [1], which did not exhibit any change in its plasma levels, in response to sleep loss (P=0.89, Wilcoxon Signed Rank Test).

## Statistical analyses

Given the large interindividual changes in miRNA levels, we calculated ratios and utilized one-sample tests. As the miRNA ratios were not normally distributed (Shapiro-Wilk test P<0.01), statistical comparisons were done using the Wilcoxon signed rank test (two-sided). For miR-127-3p and miR-132-3p, we were unable to determine qPCR CT values for one subject each, which were therefore excluded, resulting in 14 paired qPCR values for the analysis of miR-127-3p and miR-132-3p, and 15 paired qPCR values for miR-142-3p. To test the robustness of the results, we performed permutation tests (10,000 iterations, exact resampling preserving within-subject pairing; see Fig. S1). Correlations with the wake-to-sleep change in subjective stress levels at 0800h were done using the Spearman rank test. Non-parametric values are expressed as median percentage ± interquartile range (IQR), and parametric as mean ± S.E.M. percentage values. A P-value lower than 0.05 was considered significant.

# Supplemental Figures.

**Figure S1**


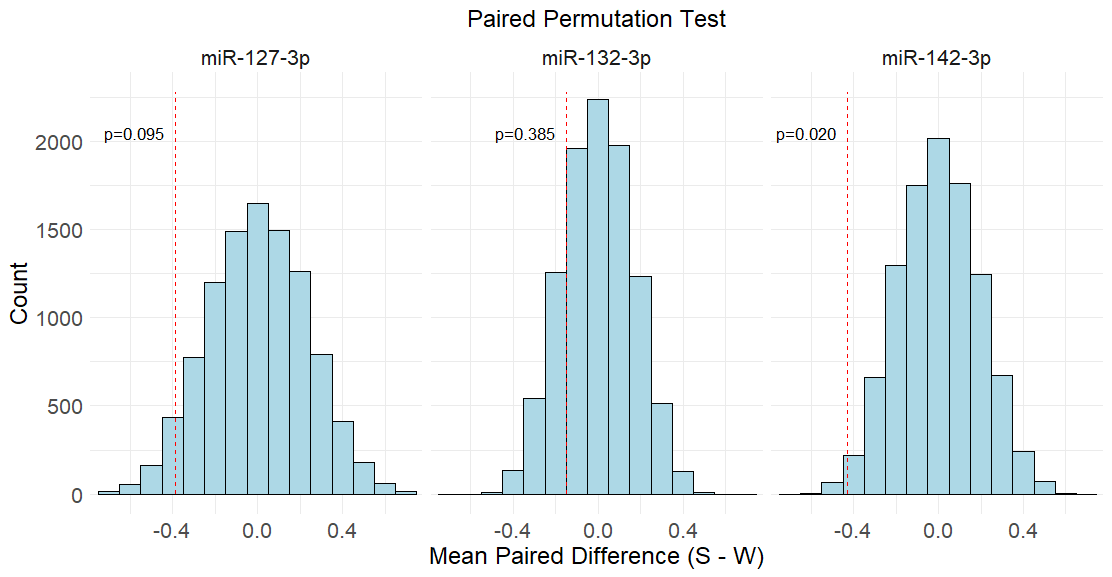


**Figure S1 Paired permutation test for miRNA expression comparing overnight wakefulness (W) and normal sleep (S).** Shows the permutation distributions of the mean paired difference (S − W) for each miRNA (miR-127-3p, miR-132-3p, miR-142-3p) over 10,000 sign-flip permutations. Each bin on the x-axis represents a range of mean differences across permuted datasets, while the y-axis displays the count of permutations within that bin. The red dashed line indicates the observed mean paired difference, and the label on the left shows the corresponding p-value. Negative mean paired difference suggests lower expression in the normal sleep (S) session compared with the overnight wakefulness (W) session.

# Supplemental Tables.

**Table S1** summarizes the differential expression of miR-127-3p, miR-132-3p and miR-142-3p across different sample types, as reported in various previous independent studies of Alzheimer’s disease (AD). Importantly, only statistically significant results have been included to focus on robust data. The data is presented as reported in the source publications, using formats such as mean ± standard deviation or median (interquartile range).

**Table S1 Overview of miR-127-3p, miR-132-3p and miR-142-3p Expression in AD Studies**

| **miRNA** | **Sample** | **N** | **Age** | **Braak stage / MMSE*** | **Regulation** | **Reference** |
| --- | --- | --- | --- | --- | --- | --- |
| miR-127-3p | CSF | 69 | 81.3 ± 7.7 | IV-VI (≥90%) | down | Burgos et al.[2] |
|  |  | 10 | 70.8 ± 12.3 | V |  | Cogswell et al.[3] |
|  | Serum | 69 | 81.3 ± 7.7 | IV-VI (≥90%) | up | Burgos et al.[2] |
|  | Brain | 41 | 79.2 ± 9.8 | — | down | Lau et al.[4] |
|  |  | 27 | 84.5 ± 8.0 | IV-VI |  | Hara et al.[5] |
| miR-142-3p | CSF | 50 | 69.52 ± 7.27 | 18.28 ± 6.40 | down | Lusardi et al.[6] |
|  | Plasma | 21 | 71.8 (69.55-74.11) | 23.20 (20.82-25.58) | down | Cosín-Tomás et al.[7] |
|  |  | 20 | 69,3 ± 6,2 | 16.5 ± 4.0 |  | Kumar et al.[8] |
|  |  | 11 | 79,6 ± 6.0 | 17.9 ± 3.5 |  | Kumar et al.[8] |
|  |  | 7 | 73.7 ± 5 | 19.85 ± 4 |  | Nagaraj et al.[9] |
|  | Serum | 47 | 65 ± 9,3 | 21 ± 5.3 | up | Denk et al[10] |
|  | Brain | 41 | 79.2 ± 9.8 | — | up | Lau et al.[4] |
|  |  | 8 | 82.9 ± 3.1 | V-VI | up | Lau et al.[4] |
| miR-132-3p | CSF | 69 | 81.3 ± 7.7 | IV-VI (≥90%) | down | Burgos et al.[2] |
|  | Serum | 66 | 72.89 ± 7.59 | MoCA: 19.89 ± 3.73 | up^**^ | Xie et al. [11] |
|  |  | 47 | 65 ± 9,3 | 21 ± 5.3 | down | Denk et al[10] |
|  | Plasma | 20 | 76.9 (range: 63-89) | 20.8 ± 8.7 | up | Sheinerman et al. [12] |
|  |  | 17 | 76 ± 7 | — | down | Walgrave et al. [13] |
|  |  | 16 | Range 60-85 | Range: 15-24 | down | Cha et al. [14] |
|  | Whole blood | 50 | 64.54±7.643 | 14.08±5.771 | down | Zhang et al. [15] |
|  |  | 24 | 76.7 ± 6.5 | 19.0 ± 3.0 | up | Hadar et al. [16] |
|  | Brain | 41 | 79,15 ± 9,77 | V-VI | down | Lau et al [4] |
|  |  | 39 | 80,4 ± 8,2 | IV-VI |  | Pichler et al [17] |
|  |  | 27 | 84.5 ± 8.0 | IV-VI |  | Hara et al.[5] |
|  |  | 16 | 81.7 ± 5.7 | VI |  | Wong et al. [18] |
|  |  | 14 | 88.2 ± 6.2 | 9.2 ± 7.5 |  | Hadar et al. [16] |
|  |  | 13 | 79.5 ± 8.7 | IV-VI |  | Zhu et al. [19] |
|  |  | 13 | 75.6 ± 1.9 | III, VI |  | Sarkar et al. [20] |
|  |  | 12 | 86.1 ± 5.8 | 16.2 ± 8.9 |  | Smith et al. [21] |
|  |  | 10 | 70.8 ± 12.3 | V |  | Cogswell et al.[3] |
|  |  | 10 | 88.6 ± 7.0 | 17.5 ± 8.1 |  | Weinberg et al. [22] |
|  |  | 10 | 72.5 ± 9.7 | — |  | Walgrave et al. [13] |
|  |  | 5 | 74,67 ± 4,67 | V-VI |  | Annese et al. [23] |
|  |  | 5 | — | — |  | Hebert et al. [24] |

* Mini-Mental State Examination (MMSE) score.

** Subjects with Mild Cognitive Impairment (MCI).

# Reference

1. Shen, Y., et al., *Identification of miR-23a as a novel microRNA normalizer for relative quantification in human uterine cervical tissues.* Experimental & Molecular Medicine, 2011. **43**(6): p. 358-366.

2. Burgos, K., et al., *Profiles of Extracellular miRNA in Cerebrospinal Fluid and Serum from Patients with Alzheimer's and Parkinson's Diseases Correlate with Disease Status and Features of Pathology.* PLOS ONE, 2014. **9**(5): p. e94839.

3. Cogswell, J.P., et al., *Identification of miRNA Changes in Alzheimer's Disease Brain and CSF Yields Putative Biomarkers and Insights into Disease Pathways.* Journal of Alzheimer's Disease, 2008. **14**(1): p. 27-41.

4. Lau, P., et al., *Alteration of the micro RNA network during the progression of Alzheimer's disease.* EMBO Molecular Medicine, 2013. **5**(10): p. 1613-1634.

5. Hara, N., et al., *Serum microRNA miR-501-3p as a potential biomarker related to the progression of Alzheimer’s disease.* Acta Neuropathologica Communications, 2017. **5**(1): p. 10.

6. Lusardi, T.A., et al., *MicroRNAs in Human Cerebrospinal Fluid as Biomarkers for Alzheimer’s Disease.* Journal of Alzheimer's Disease, 2016. **55**(3): p. 1223-1233.

7. Cosín-Tomás, M., et al., *Plasma miR-34a-5p and miR-545-3p as Early Biomarkers of Alzheimer’s Disease: Potential and Limitations.* Molecular Neurobiology, 2017. **54**(7): p. 5550-5562.

8. Kumar, P., et al., *Circulating miRNA Biomarkers for Alzheimer's Disease.* PLoS ONE, 2013. **8**(7): p. e69807.

9. Nagaraj, S., et al., *Profile of 6 microRNA in blood plasma distinguish early stage Alzheimer’s disease patients from non-demented subjects.* Oncotarget, 2017. **8**(10): p. 16122-16143.

10. Denk, J., et al., *Specific serum and CSF microRNA profiles distinguish sporadic behavioural variant of frontotemporal dementia compared with Alzheimer patients and cognitively healthy controls.* PLOS ONE, 2018. **13**(5): p. e0197329.

11. Xie, B., et al., *Serum miR-206 and miR-132 as Potential Circulating Biomarkers for Mild Cognitive Impairment.* Journal of Alzheimer’s Disease, 2015. **45**(3): p. 721-731.

12. Sheinerman, K.S., et al., *Plasma microRNA biomarkers for detection of mild cognitive impairment.* Aging, 2012. **4**(9): p. 590-605.

13. Walgrave, H., et al., *Restoring miR-132 expression rescues adult hippocampal neurogenesis and memory deficits in Alzheimer’s disease.* Cell Stem Cell, 2021. **28**(10): p. 1805-1821.e8.

14. Cha, D.J., et al., *miR-212 and miR-132 Are Downregulated in Neurally Derived Plasma Exosomes of Alzheimer’s Patients.* Frontiers in Neuroscience, 2019. **13**.

15. Zhang, H., et al., *The diagnostic value of blood miR-132-3p level in patients with Alzheimer's disease.* preprint, 2024.

16. Hadar, A., et al., *SIRT1, miR-132 and miR-212 link human longevity to Alzheimer’s Disease.* Scientific Reports, 2018. **8**(1): p. 8465.

17. Pichler, S., et al., *The miRNome of Alzheimer's disease: consistent downregulation of the miR-132/212 cluster.* Neurobiology of Aging, 2017. **50**: p. 167.e1-167.e10.

18. Wong, H.-K.A., et al., *De-repression of FOXO3a death axis by microRNA-132 and -212 causes neuronal apoptosis in Alzheimer's disease.* Human Molecular Genetics, 2013. **22**(15): p. 3077-3092.

19. Zhu, Q.-B., et al., *MicroRNA-132 and early growth response-1 in nucleus basalis of Meynert during the course of Alzheimer’s disease.* Brain, 2016. **139**(3): p. 908-921.

20. Sarkar, S., et al., *Expression of microRNA-34a in Alzheimer's disease brain targets genes linked to synaptic plasticity, energy metabolism, and resting state network activity.* Brain Research, 2016. **1646**: p. 139-151.

21. Smith, P.Y., et al., *miR-132/212 deficiency impairs tau metabolism and promotes pathological aggregation in vivo.* Human Molecular Genetics, 2015. **24**(23): p. 6721-6735.

22. Weinberg, R.B., E.J. Mufson, and S.E. Counts, *Evidence for a neuroprotective microRNA pathway in amnestic mild cognitive impairment.* Frontiers in Neuroscience, 2015. **9**.

23. Annese, A., et al., *Whole transcriptome profiling of Late-Onset Alzheimer’s Disease patients provides insights into the molecular changes involved in the disease.* Scientific Reports, 2018. **8**(1): p. 4282.

24. Hébert, S.S., et al., *A Study of Small RNAs from Cerebral Neocortex of Pathology-Verified Alzheimer's Disease, Dementia with Lewy Bodies, Hippocampal Sclerosis, Frontotemporal Lobar Dementia, and Non-Demented Human Controls.* Journal of Alzheimer’s Disease, 2013. **35**(2): p. 335-348.
